# Association between C3435T polymorphism of *MDR1* gene and the incidence of drug-resistant epilepsy in the population of Polish children

**DOI:** 10.1186/s12993-016-0106-z

**Published:** 2016-07-08

**Authors:** Mariusz Stasiołek, Hanna Romanowicz, Katarzyna Połatyńska, Maciej Chamielec, Dominik Skalski, Marianna Makowska, Beata Smolarz

**Affiliations:** Department of Neurology, Polish Mother’s Memorial Hospital-Research Institute, Rzgowska 281/289, 93-338 Lodz, Poland; Laboratory of Cancer Genetics, Department of Clinical Pathomorphology, Polish Mother’s Memorial Hospital-Research Institute, Rzgowska 281/289, 93-338 Lodz, Poland; Department of Neurology, Regional Hospital Dr. Charles Jonscher, Milionowa 14, 93-113 Lodz, Poland; Department of Neurology, Charité-Universitätsmedizin Berlin, 10117 Berlin, Germany

**Keywords:** *MDR1*, Drug-resistant epilepsy, Drug-responsive epilepsy, Gene polymorphism, Children

## Abstract

**Purpose:**

Epilepsy is a disease of neurological character. Approximately one third of epileptic patients demonstrate a drug-resistant phenotype, which is associated with the development of drug-resistant epilepsy. The multidrug resistance protein 1 and glycoprotein P, encoded by *MDR1*, play a significant role in the transmembrane transport of anti-epileptic agents. Single nucleotide polymorphism C3435T (rs1045642) within *MDR1* gene may be associated with an increased expression of P-gp which affects the levels of antiepileptic drugs in plasma. The presented studies analysed the association between C3435T polymorphism of *MDR1* gene and the incidence of drug-resistant epilepsy in the population of Polish children.

**Methods:**

C3435T polymorphism of *MDR1* gene was analysed by the high resolution melting technique in a group of patients with drug-resistant (n = 106) and drug-responsive epilepsy (n = 67), as well as in non-epileptic children (n = 98) hospitalised at the Department of Neurology, Polish Mother’s Memorial Hospital in Lodz. Genotype and allele distributions were evaluated and their compatibility with the Hardy–Weinberg distribution was assessed by means of the χ^2^ test. Genotype and allele evaluation, regarding their relationship with a given feature, was supported by an analysis of odds ratio and 95 % confidence interval, calculated according to the logistic regression model.

**Results:**

An association was observed between the incidence rate of DRE and the presence of C allele in C3435T polymorphism of *MDR1* gene, which may enhance the risk of the disease. The T allele may then play a protective role. No differences were found in the studied groups, regarding either genotype or allele distribution in reference to patient’s gender or concomitant diseases.

**Conclusion:**

Following the obtained results, C3435T polymorphism of *MDR1* gene may be connected with the incidence of drug-resistant epilepsy in the population of Polish children.

ISRCTN ISRCTN73824458. Registered 28th September 2014.

## Background

Epilepsy is one of the most frequent diseases of the nervous system, with prevalence rate estimated at approx. 1 % of the population in the world. The incidence of epilepsy is slightly higher in men than in women, while presenting significantly higher rates in children and in subjects above 65 years. The average incidence rate in the world population amounts to 50–70/100,000/year. In Poland, the number of affected subjects approaches 400,000 with the incidence of 7/1000 inhabitants [1]. For comparison, in the Asian populations, e.g., in Japan, the incidence is 1.5/1000 and in India, 5.59/1000 [1, 2].

The basic treatment of epilepsy consists of a wide range of pharmacological substances—anticonvulsant medications. However, despite the constant development of new anticonvulsants with various molecular mechanisms of action, the fully effective pharmacological treatment of epilepsy has not yet been reached. In some of the patients, so-called, DRE may occur, as a serious clinical problem.

There are two main theories to explain the phenomenon of drug-resistance. The hypothesis of transporters assumes that the expression or function of multidrug transporters gets enhanced in the brain, thus suppressing the access of antiepileptic drugs to the central nervous system. The other hypothesis is based on the assumption that epilepsy-related changes induce lower sensitivity to anticonvulsant [3].

Despite the increasing knowledge of suspected risk factors of DRE, it still unclear why the responsiveness to pharmacological treatment may vary significantly between epilepsy patients with supposedly similar clinical manifestations of the disease. The suggested determinants of DRE include genetic factors and varying pharmacodynamic and pharmacokinetic features of administered drugs [4–7].

The to-date’s literature data indicate a significant role of MDR 1 and P-gp, encoded by *MDR1,* in the transmembrane transport of anti-epileptic agents [8–10]. The C3435T polymorphism (rs1045642) of *MDR1* gene has been suggested as one of the genetic factors underlying DRE phenomenon [8–13]. It has been demonstrated that C3435T polymorphism determines the modified expression of *MDR1* gene and thus the activity of P-gp, which plays a significant role in the transmembrane drug transport [10]. It has been demonstrated that P-gp participates in the transport of phenobarbital, carbamazepine, phenytoin, lamotrigine, felbamate and gabapentin through the blood–brain barrier, as well as through the cellular membranes of astrocytes and neurons in an epileptic focus. The results of the studies indicate participation of P-gp in the pathogenesis of DRE [10].

The *MDR1* gene polymorphism, while structuring the variable expression of P-gp, may then control the efficacy of anti-epileptic therapy. An increased expression of this gene leads to higher volumes of P-gp in the endothelial cells of the blood–brain barrier and in astrocytes. This process results in decreasing the parenchymal concentrations of drugs, despite achieved therapeutic level in blood [10, 13]. Importantly, in patients with DRE, evaluated by van Vliet et al. and Lazarowski et al., the homozygotic CC genotype was associated with increased P-gp expression, which affected the concentration levels of anti-epileptic drugs in plasma [10, 13].

However, the currently available literature data lack an unequivocal response to the question about the exact role of the *MDR1* gene C3435T polymorphism in the development of DRE in children [12–17].

The goal of the study was to analyse the association between C3435T polymorphism of *MDR1* gene and DRE in the population of Polish children.

## Methods

### Patients

Whole blood samples (1 ml), used as material for molecular studies, were collected from children with DRE (n = 106) and drug-responsive (n = 67) epilepsy, as well as from non-epileptic children (n = 98), hospitalised at the Department of Neurology, Polish Mother’s Memorial Hospital, Research Institute, in the years 2010–2016. The group of children with DRE included 52 boys and 54 girls and the group of therapy responders included 35 boys and 32 girls, while the group of non-epileptic children comprised 52 boys and 46 girls. The demographic parameters of the study groups were not significantly different (see Table 1). All of the studied individuals, patients and controls were Caucasians from the same ethnic and geographical origins, living in the Lodz region of central Poland.

**Table 1:** The number and age of the patients submitted to *MDR1* gene polymorphism studies

Children with drug-resistant epilepsy (n = 106)	Children with drug-responsive epilepsy (n = 67)	Healthy children (n = 98)
Age (range) 1–15 years	Age (range) 1–16 years	Age (range) 4–14 years
Age (mean) 8.5 ± 4.84	Age (mean) 8.2 ± 4.019	Age (mean) 8.3 ± 4.64

All the epilepsy patients were subjected to pharmacotherapy. The administered medicinal agents included: CBZ, topiramat, OXC, GBP, LTG and LEV—all in various doses and configurations, depending on patient’s health status. A formal consent was obtained from the Bioethical Committee of the Institute of the Polish Mother’s Memorial Hospital in Lodz (decision of 15th December 2010). Genetic studies of *MDR1* gene C3435T polymorphism were performed at the Laboratory of Cancer Genetics, Department of Clinical Pathomorphology, Institute of the Polish Mother’s Memorial Hospital in Lodz.

### HRM analyses

Genomic DNA was prepared, using DNeasy Blood & Tissue Kit (Qiagen GmbH, Hilden, Germany) according to the manufacturer instruction. PCR products for the analysed variants were analysed by HRM analysis. Real-time PCR cycling and conditions and primers for HRM analysis of all the examined MDR1 SNPs are summarized in Table 2. The analysis of the SNPs was performed with support of a Light Cycler^®^ 480 High Resolution Melting Master Kit (Roche, Mannheim, Germany), according to the manufacturer’s recommendations. A non-template control contained water, instead of genomic DNA, as a negative control. Additionally, positive controls (DNA samples with known genotype) were employed in each run of HRM analysis. PCR amplification was performed in a LightCycler^®^ 96 (Roche, Mannheim, Germany) Thermocycler. The collected data were analysed, using the LightCycler^®^ 96 software version SW 1.1 (Roche, Mannheim, Germany).

**Table 2:** Primer sequences and conditions for real-time PCR and the following HRM analysis of the examined *MDR1* C3435T

Target	Primers (5′–3′)	PCR cycling (40 cycles)			HRM
		Denaturation	Annealing	Extension	
rs1045642	Forward: TCCTGAAGTTGATCTGTGAAC Reverse: AGTGACTCGATGAAGGCA	30 s for 95 °C	30 s for 58 °C	30 s for 72 °C	75–90 °C

### Statistical analysis

Genotype and allele distributions were evaluated and their compatibility with HWE distribution was assessed by means of the χ^2^ test. Differences between distributions in particular groups were evaluated also by the χ^2^ test. All multivariate models were adjusted for age and use of variety of drugs. *T* test (for normal distribution) or Mann–Whitney test (for non-normal distribution) was used to compare each parameter between two groups. An ANOVA test was used to identify parameters that would make significant differences between more than two groups; Scheffe’s test was then used to test the significance of difference in each identified parameter between any two groups. Genotype and allele evaluation, regarding their relationship with a given feature, e.g., a risk for disease, was supported by an analysis of OR and 95 % CI, calculated according to the logistic regression model. The wild type of genotype and allele was a reference group. The Statistica v. 7.0 software package (StatSoft, Tulsa, OK, USA) was used.

## Results

It was demonstrated that the *MDR1* gene C3435T polymorphism may be associated with the incidence of DRE in Polish children.

See Table 3 for the distribution of genotypes and the incidence of alleles in the group of DRE and in the group of non-epileptic children. Statistically significant differences were demonstrated in genotype distribution between the study groups (*p* < 0.05). The CC genotype occurred in the patients with statistically significantly higher frequency, while the TT homozygote was found with statistically significantly lower frequency vs. the group of healthy controls (*p* < 0.05). It was demonstrated that the C allele in those children may be a risk factor for DRE, unlike the T allele which, by contrast, may play a protective role (OR 0.53; 95 % CI, 0.33–0.85; *p* = 0.012).

**Table 3:** Distributions of TT, CT and CC genotypes and T and C alleles in C3435T polymorphism of *MDR1* gene in the group of children with drug-resistant epilepsy and in the group of healthy children

	Children with drug-resistant epilepsy (n = 106)		Healthy children (n = 98)		OR (95 % CI^a^)^b^	*p* ^c^
	Number	(%)	Number	(%)		
CC	35	33	16	16	1.00 Ref.^d^	
CT	39	37	37	38	0.48 (0.23–1.01)	0.079
TT	32	30	45	46	0.33 (0.15–0.68)	0.005
C	109	51	69	35	1.00 Ref.	
T	103	49	127	65	0.53 (0.33–0.85)	0.012

^a^Crude odds ratio (OR), 95 % CI confidence interval at 95 %

^b^Adjusted for age and use of variety of drugs

^c^for the test of χ^2^ conformity with the distribution predicted by the Hardy–Weinberg law

^d^Reference group—wild type of allele

The observed genotype frequency of *MDR1* C3435T in the controls group were in agreement with HWE (*p* > 0.05). In case of the C3435T polymorphism of *MDR1* gene the distribution of the genotypes in the patients differed significantly from one expected from HWE (*p* < 0.05). It is caused by the very low abundance of the *MDR1* TT genotype in the examined Polish population.

Similar results were observed when the group of epileptic non-responders was compared with the responders (see Table 4). The CC homozygote in the patients with DRE occurred with a statistically significantly higher frequency (33 %), while the TT homozygote demonstrated a statistically significantly lower frequency (30 %) vs. the group of children with therapy-responding epilepsy (CC-24 %, TT-49 %). The C allele in the patients with DRE occurred with a statistically significantly higher frequency (51 %), while the T allele demonstrated a statistically significantly lower frequency (49 %) vs. the group of children therapy-responding epilepsy (C-37 %, T-63 %). The C allele in those children may be a risk factor for DRE. The T allele may then play a protective role (OR 0.56 95 % CI 0.36–0.87; *p* = 0.014).

**Table 4:** Distributions of TT, CT and CC genotypes and T and C alleles in C3435T polymorphism of *MDR1* gene in the group of children with drug-resistant epilepsy and in the group of drug-responsive epilepsy

	Children with drug-resistant epilepsy (n = 106)		Children with drug-responsive epilepsy (n = 67)		OR (95 % CI^a^)^b^	*p* ^c^
	Number	(%)	Number	(%)		
CC	35	33	16	24	1.00 Ref.^d^	
CT	39	37	18	27	0.99 (0.44–2.23)	0.862
TT	32	30	33	49	0.44 (0.21–0.95)	0.036
C	109	51	50	37	1.00 Ref.	
T	103	49	84	63	0.56 (0.36–0.87)	0.014

^a^Analysis of odds ratio (*OR* odds ratio, *PU* Confidence Interval 95 %)

^b^Adjusted for age and use of variety of drugs

^c^For the test of χ^2^ conformity with the distribution predicted by the Hardy–Weinberg law

^d^Reference group—wild type of allele

No statistically significant differences were demonstrated in the distribution of genotypes between the group of responders and healthy controls (*p* > 0.05) (see Table 5).

**Table 5:** Distributions of TT, CT and CC genotypes and T and C alleles in C3435T polymorphism of *MDR1* gene in the group of children with drug-responsive epilepsy and in the group of healthy children

	Children with drug-responsive epilepsy (n = 67)		Healthy children (n = 98)		OR (95 % CI^a^)^b^	*p* ^c^
	Number	(%)	Number	(%)		
CC	16	24	16	16	1.00 Ref.^d^	
CT	18	27	37	38	0.48 (0.19–1.18)	0.172
TT	33	49	45	46	0.73 (0.32–1.67)	0.596
C	50	37	69	35	1.00 Ref.	
T	84	63	127	65	0.92 (0.58–1.44)	0.777

^a^Analysis of odds ratio (*OR* odds ratio, *PU* Confidence Interval 95 %)

^b^Adjusted for age and use of variety of drugs

^c^For the test of χ^2^ conformity with the distribution predicted by the Hardy–Weinberg law

^d^Reference group—wild type of allele

No differences were found in the study groups, either in genotype or allele distribution in reference to patient’s gender or concomitant diseases (*p* > 0.05).

## Discussion

The *MDR1* gene polymorphism, while structuring the variable expression of P-gp, may then control the efficacy of anti-epileptic therapy. An increased expression of this gene leads to higher volumes of P-gp in the endothelial cells of the blood–brain barrier and in astrocytes. This process results in decreased parenchymal concentrations of drugs, despite their achieved therapeutic levels in blood [18, 19]. It should be emphasised that the majority of data from world literature concerns SNPs of *MDR1*-C3435T.

In the presented study, a genetic evaluation was performed of the *MDR1* gene C3435T polymorphism in order to define its association with DRE in the population of Polish children. This polymorphism was selected on the basis of the earlier mentioned literature data, which are suggestive of its correlations with DRE [8–11]. Studies of this type have been rather scarce in the world perspective, what raised our interest in this challenging issue.

The polymorphisms of *MDR1* gene, including C3435T polymorphism, were analysed by Alpman et al. [17] in a group of 39 children with DRE vs. a control group (n = 92). A conclusion was drawn that the polymorphisms of *MDR1* gene were not associated with multidrug resistance but that CC3435/GG2677 genotypes might affect the efficacy of administered therapies in cases of DRE.

Shaheen et al. [15] studied the functional importance of C3435T polymorphism in a population of epileptic children in the southern India. The risk of non-responsiveness to therapy was much higher in patients, being the carriers of TT genotype vs. CC homozygotic patients. The researchers suggested that C3435T polymorphism could be related to DRE, however, further studies in different ethnic populations are necessary to explain the role of the polymorphism in DRE patients.

However, there are also reports which do not confirm the relationship between C3435T polymorphism of *MDR1* gene and DRE in children [12, 14, 16].

In Poland, two reports have been published on this issue, describing a study of C3435T polymorphism in a group of children from the Silesian region [20, 21]. The obtained results suggest no relationship between the occurrence of DRE and the above-mentioned polymorphism of *MDR1* gene in the population of Polish children. Following the authors’ suggestions, further genetic studies are necessary to better understand the causes of DRE, which may help in developing a more effective therapy.

In contrast to those reports, in this reported study, the influence of C allele on the risk of DRE in children was found and confirmed. An increased incidence rate of CC genotype was found in the group of non-responders to therapy vs. both the responders and healthy controls. A decreased incidence rate of TT homozygote was found in the group of non-responders to therapy vs. both the responders and healthy controls. It suggests that the T allele may play a protective role.

It is possible that the presence of *MDR1* allele remains in a linkage disequilibrium with another, so far unknown, mutation, which may be important, regarding P-gp concentrations in plasma.

A number of hypotheses try to explain the relationship between the *MDR1* C3435T polymorphism and the activity of P-gp. The first hypothesis assumes some conjugation of 3435T allele, which correlates with decreased transporter activity, with G2677 (A, T) changes, leading to modifications in the sequence of amino acids (Val > Ser, Val > Thr). A consequence of these changes is different stability and activity of this protein [22, 23].

Since this conjugation is not complete and its degree differs in particular populations, the above-mentioned correlation was not observed in all the reported studies. The presence of other, missense type, polymorphisms, e.g., C1236T, may be an additional worrying factor [22]. Another theory assumes a correlation of the C3435T polymorphism with other, still not identified changes in the regulatory domains of this gene, which may affect its expression levels. However, there is no reliable literature data to support this hypothesis.

The obtained results demonstrate a possibility of a relationship between C3435T polymorphism and the incidence rate of DRE in the population of the Polish children. However, it requires further studies on much larger study groups. Still, one cannot exclude a possible influence of the studied polymorphism on epilepsy development in result of combined effects with other factors. For the process of epilepsy development is complicated and demanding a complex participation of multiple factors.

Drawing conclusions from obtained data should be done with a substantial level of caution due to the limitations affecting this study: test group and controls may be quantitatively unsatisfactory, SNPs linkage disequilibrium was not considered, circulating P-gp levels in patients were unknown and the relation between SNP C3435T in *MDR1* and P-gp expression has not been stated. Among the abundance of genetic polymorphisms in *MDR1* gene only one polymorphism of *MDR1* was analysed without respect to linkage disequilibrium, which is a crucial feature of population genetics. A joint analysis of SNP C3435T of *MDR1* and other SNPs in *MDR1* could shed a new light on the role of SNPs in DRE.

## Abbreviations

CBZ: carbamazepine
CI: confidence interval
DRE: drug-resistant epilepsy
GBP: gabapentin
HRM: high resolution melting
HWE: Hardy-Weinberg equilibrium
LEV: levetiracetam
LTG: lamotrigine
MDR1: multidrug resistance protein 1
OR: odds ratio
OXC: oxcarbazepine
P-gp: P-glycoprotein
SNP: single nucleotide polymorphism
